# Global burden and cross-country inequalities in urinary tumors from 1990 to 2021 and predicted incidence changes to 2046

**DOI:** 10.1186/s40779-025-00599-y

**Published:** 2025-03-17

**Authors:** De-Chao Feng, Deng-Xiong Li, Rui-Cheng Wu, Jie Wang, Yu-Han Xiao, Koo Han Yoo, Xing Ye, Wu-Ran Wei, De-Pei Kong, Zhou-Ting Tuo

**Affiliations:** 1 Department of Urology, Institute of Urology, West China Hospital, Sichuan University, Chengdu, 610041 China; 2 Division of Surgery and Interventional Science, University College London, London, WC1E 6BT UK; 3 Department of Rehabilitation, the Affiliated Hospital of Southwest Medical University, Luzhou, 646000 Sichuan China; 4 Department of Urology, Kyung Hee University, Seoul, 446 701 South Korea; 5 Cedars-Sinai Medical Center, Los Angeles, CA 90048 USA; 6 Department of Urology, the Second Affiliated Hospital of Anhui Medical University, Hefei, 230601 China

**Keywords:** Disability-adjusted life-years (DALYs), Health inequality, Global burden of disease, Urinary tumors

Dear Editor,

The global population of individuals aged 65 and older is projected to reach 1.6 billion by 2050 [1]. Given that urinary tumors, such as bladder cancer (BCa), kidney cancer (KCa), and prostate cancer (PCa), are more common in older adults, the burden on the healthcare system is increasing [2]. Recently, Zi et al. [3] conducted a comprehensive assessment of the global burden of 6 urinary diseases from 1990 to 2021, based on the Global Burden of Diseases, Injuries, and Risk Factors Study 2021. This study describes the burden and age-sex distribution of 4 urinary tumors, including BCa, KCa, PCa, and testicular cancer (TCa), and further analyzes cross-country inequalities and projects future incidence rates globally up to 2046 for all these cancers [4, 5]. Our analyses and visualizations were conducted using the World Health Organization Health Equity Assessment Toolkit and R software (version 4.2.3).

For the 4 malignancies under investigation, there were notable differences in age-standardized rates (ASR) of disability-adjusted life-years (DALYs) across 204 countries and territories in 2021 (Additional file 1: Tables S1–3). For individuals with BCa, KCa, and PCa, the DALYs rate increased steadily with age in 2021 (Fig. 1a–c). Notably, for BCa and KCa, the DALYs rate was consistently greater in men than in women across all age categories. In contrast, for TCa, there was no discernible relationship between the DALYs rate and age, with the greatest rates observed in the 25 − 29 years age group (Fig. 1d). Our analysis of health inequality distribution for BCa, KCa, PCa, and TCa reveals notable trends over time, based on the Slope Index of Inequality (SII) and Concentration Index (CI). For BCa, the SII increased from 76.07 in 1990 to 110.57 in 2021, indicating a widening disparity in the BCa burden between high and low socio-demographic index (SDI) countries (Fig. 1e; Additional file 1: Table S4). The CI for BCa also rose slightly, from 0.36 in 1990 to 0.38 in 2021, reflecting a modest increase in the concentration of BCa burden in higher SDI countries (Fig. 1f; Additional file 1: Table S4). Similarly, for KCa, the SII increased from 57.31 in 1990 to 105.97 in 2021, demonstrating a comparable trend of growing inequality (Fig. 1g; Additional file 1: Table S4). The CI for KCa showed a slight decrease, from 0.41 in 1990 to 0.39 in 2021, indicating a minor reduction in the concentration of KCa burden in higher SDI countries (Fig. 1h; Additional file 1: Table S4). For PCa, the SII increased substantially from 329.90 in 1990 to 544.03 in 2021, signaling a significant exacerbation of inequality in PCa over time (Fig. 1i; Additional file 1: Table S4). The CI for PCa decreased from 0.44 in 1990 to 0.31 in 2021, suggesting a reduction in the concentration of PCa burden in higher SDI countries, which may reflect a shift in global PCa trends (Fig. 1j; Additional file 1: Table S4). In contrast, for TCa, the SII decreased from 14.45 in 1990 to 7.32 in 2021, indicating a reduction in inequality, with a smaller gap between high and low SDI countries (Fig. 1k; Additional file 1: Table S4). The CI for TCa showed a marked decline from 0.21 in 1990 to 0.06 in 2021, reflecting a significant reduction in the concentration of TCa burden in higher SDI countries, suggesting improved equality in the distribution of TCa outcomes (Fig. 1l; Additional file 1: Table S4). For BCa, KCa, and PCa, SDI-related health disparities have exacerbated over time, with increasing gaps between high and low SDI countries. These cancers have become more concentrated in higher SDI nations, especially for PCa. In contrast, TCa shows a significant improvement, with both a decrease in the SII and CI, suggesting a reduction in health inequality related to TCa over the same period. These findings highlight the growing burden of BCa, KCa, and PCa in higher SDI countries, while TCa outcomes have become more equally distributed across SDI groups.

**Fig. 1:**
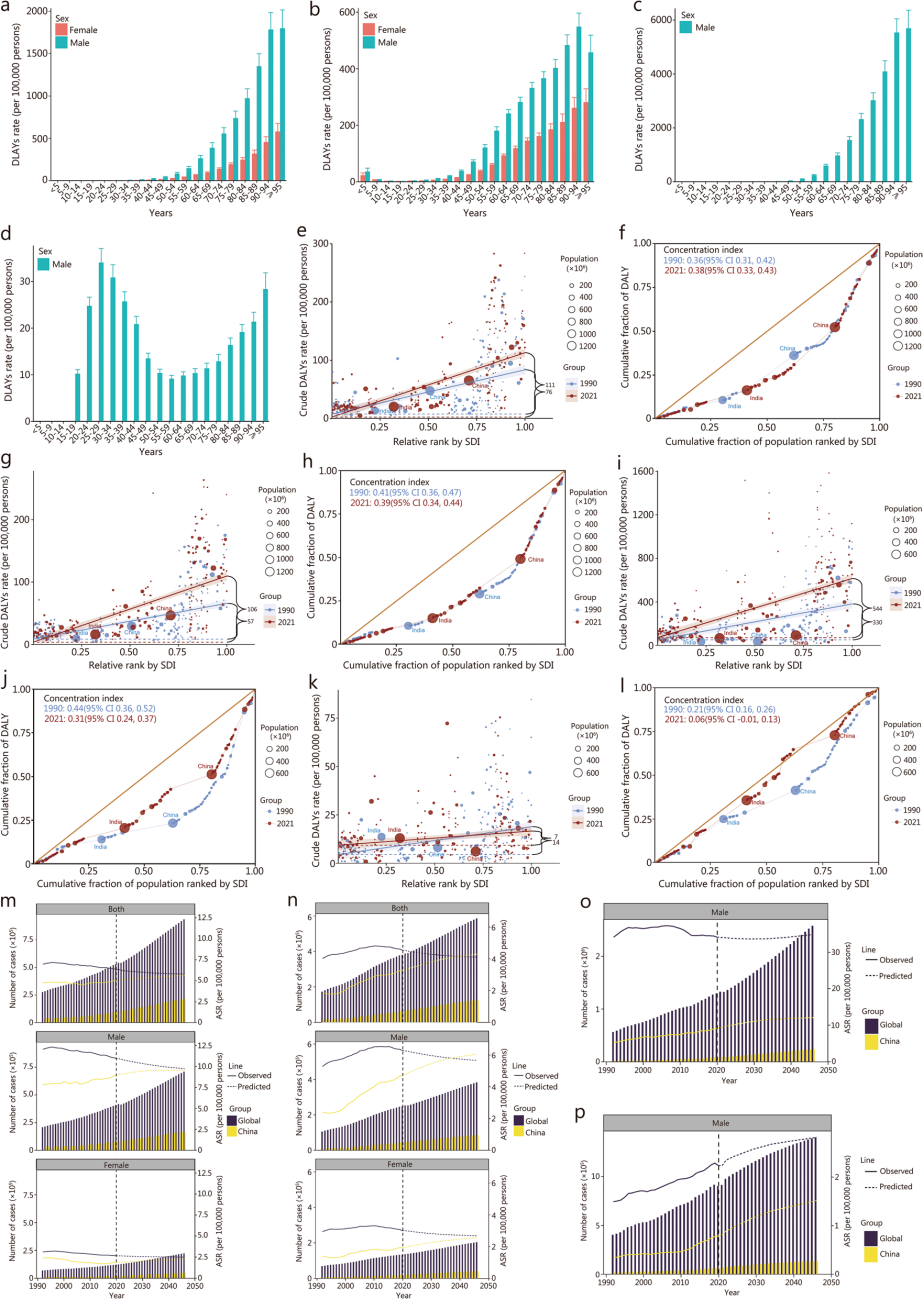
Global burden and cross-country inequalities in 4 urinary tumors from 1990 to 2021 and predicted incidence changes to 2046. Age and sex distribution of global DLAYs rate of BCa (**a**), KCa (**b**), PCa (**c**), and TCa (**d**) in 2021. Health inequality regression curves and concentration curves for the DALYs of BCa (**e** and** f**), KCa (**g** and** h**), PCa (**i** and** j**), and TCa (**k** and** l**) worldwide from 1990 to 2021. The global and China trends in ASR and case number of BCa (**m**), KCa (**n**), PCa (**o**), and TCa (**p**) between 1990 and 2021, and predicted changes to 2046. For Fig. 1 **m**–**p**, the bar graph represents the case number of tumor patients and the line graph represents the ASR rate in global and China. BCa bladder cancer, KCa kidney cancer, PCa prostate cancer, TCa testicular cancer, ASR age-standardized rate, DALYs disability-adjusted life-years, SDI sociodemographic index

Using the Norpred model, we predicted the global ASR of incidence for 4 urinary tumors across different regions and populations. From 1992 to 2021, the ASR of incidence for BCa, KCa, PCa, and TCa increased significantly worldwide, while the ASR of incidence for the other 3 tumors showed a slowing trend except TCa (Fig. 1m–p; Additional file 1: Table S5). According to the forecast, from 2022 to 2046, the number of cases for all 4 cancers is expected to rise significantly globally. However, with the exception of TCa, the growth in the ASR of incidence for the other 3 cancers is projected to slow considerably. Notably, the ASR of incidence for all 4 tumors in China is anticipated to show a marked upward trend between 2022 and 2046. These findings align with predictions of an aging population by 2050 and suggest that age may become a primary determinant of cancer incidence in the future [1].

Our study highlights the significant challenges in treating urinary malignancies, revealing an increasing incidence and substantial regional disparities in the distribution of these conditions. These findings offer crucial insights for developing public health policies and ensuring the judicious allocation of medical resources. Despite the limitations imposed by data sources and financial constraints, the study's strengths lie in its comprehensive use of data across a wide geographical area and extended period. Projections for the future indicate an increasing ASR of incidence of these malignancies, with aging being a major contributing factor, emphasizing the growing need for targeted interventions and resource allocation.

## Supplementary Information

**Additional file 1:** Materials and methods. **Table S1** Age-standardized rate (ASR) of disability-adjusted Life years (DALYs) for bladder cancer (BCa) by sex for all countries in 2021. **Table S2** Age-standardized rate (ASR) of disability-adjusted Life years (DALYs) for kidney cancer (KCa) by sex for all countries in 2021. **Table S3** Age-standardized rate (ASR) of disability-adjusted Life years (DALYs) for prostate cancer (PCa) and testicular cancer (TCa) for all countries in 2021. **Table S4** Summary indicators of sociodemographic index (SDI) related inequality in age-standardized rate (ASR) of disability-adjusted Life years (DALYs) for four urinary tumors worldwide in 1990 and 2021. **Table S5** The global trends in age-standardized rate (ASR) and case number of incidence for four urinary tumors between 1992 and 2021, and predicted changes to 2046.

## Data Availability

The data used in this study can be derived from the GBD 2021 (Available at: https://ghdx.healthdata.org/gbd-2021).

## Abbreviations

ASR: Age-standardized rate
BCa: Bladder cancer
CI: Concentration index
DALYs: Disability-adjusted life-years
KCa: Kidney cancer
PCa: Prostate cancer
SDI: Sociodemographic index
SII: Slope index of inequality
TCa: Testicular cancer
