# Supplementary material for: Global burden and cross-country inequalities in urinary tumors from 1990 to 2021 and predicted incidence changes to 2046
Source: Mil Med Res. 2025 Mar 17;12:12. doi: 10.1186/s40779-025-00599-y (PMC11912679; doi:10.1186/s40779-025-00599-y)
Supplement: Supplementary file 1 — Additional file 1: Materials and methods. Table S1 Age-standardized rate (ASR) of disability-adjusted Life years (DALYs) for bladder cancer (BCa) by sex for all countries in 2021. Table S2 Age-standardized rate (ASR) of disability-adjusted Life years (DALYs) for kidney cancer (KCa) by sex for all countries in 2021. Table S3 Age-standardized rate (ASR) of disability-adjusted Life years (DALYs) for prostate cancer (PCa) and testicular cancer (TCa) for all countries in 2021. Table S4 Summary indicators of sociodemographic index (SDI) related inequality in age-standardized rate (ASR) of disability-adjusted Life years (DALYs) for four urinary tumors worldwide in 1990 and 2021. Table S5 The global trends in age-standardized rate (ASR) and case number of incidence for four urinary tumors between 1992 and 2021, and predicted changes to 2046. [file 40779_2025_599_MOESM1_ESM.pdf]

Materials and methods

Data source

In the present study, the estimates and their 95% uncertainty intervals (UI) for the disability-adjusted life years (DALYs) of bladder cancer (BCa), kidney cancer (KCa), prostate cancer (PCa), and testicular cancer (TCa) were obtained from the GBD 2021 database [1, 2]. Additionally, we retrieved global socio-demographic index (SDI) data from this database, which quantifies the socio-demographic development level of a country or region based on factors such as income, education, and fertility status [3].

Burden description

The age-standardized rate (ASR) of DALYs for urinary tumors was presented at the national level for 2021 [4]. Additionally, the age and sex distribution of the burden of these diseases were also examined.

Cross-country inequality analysis

We calculate the slope index of inequality and the concentration index according to the standard health equity analytic methods recommended by the World Health Organization (https://www.who.int/data/inequality-monitor). These two indicators aim to quantify the distributive inequality of the burden of four urinary tumors across countries [5]. The slope index of inequality was defined by the midpoint of the population accumulation range ranked by SDI, which is calculated by regression of the DALYs of the population of all ages on the SDI-related relative position scale. The concentration index was calculated by numerically integrating the area under the Lorenz concentration curve, which was fitted using the cumulative fraction of DALYs and cumulative relative distribution of population ranked by SDI [6].

Norpred predictive analysis

We obtained standard population data and projected population data in the GBD 2021 (https://ghdx.healthdata.org/gbd-2021). A log-linear age-period-cohort model leveling off exponential growth and limiting linear trend projection was used to predict the new case number and incidence rates through 2046 [7, 8]. The prediction was implemented using the NORDPRED

package (in R software), which has been determined to perform well in projecting current trends in cancer into the future [9].

Statistical analysis

The DALYs rate and incidence were expressed as the estimate per 100,000 population and its 95% uncertainty interval (UI). Slope index of inequality and concentration index are used to evaluate the distribution characteristics and imbalance degree of health status. The calculation of inclination index uses the mean, standard deviation and deviation of sample data to describe the skewness of health status distribution. The concentration index is used to quantify the uneven distribution of health status among different groups. The closer the index value is to 1, the more concentrated the distribution is, and the closer it is to -1, the more dispersed it is. In order to quantify the statistical uncertainty of these indexes, we calculated their respective 95% confidence intervals (CI). Our analyses and visualizations were performed using the World Health Organization Health Equity Assessment Toolkit and R software (v.4.2.3).

References

1. GBD 2021 Diseases and Injuries Collaborators. Global incidence, prevalence, years lived with disability (YLDs), disability-adjusted life-years (DALYs), and healthy life expectancy (HALE) for 371 diseases and injuries in 204 countries and territories and 811 subnational locations, 1990-2021: a systematic analysis for the Global Burden of Disease Study 2021. Lancet. 2024;403(10440):2133-61.
2. GBD 2021 US Burden of Disease Collaborators. The burden of diseases, injuries, and risk factors by state in the USA, 1990-2021: a systematic analysis for the Global Burden of Disease Study 2021. Lancet. 2024;404(10469):2314-40.
3. GBD 2021 Risk Factors Collaborators. Global burden and strength of evidence for 88 risk factors in 204 countries and 811 subnational locations, 1990-2021: a systematic analysis for the Global Burden of Disease Study 2021. Lancet. 2024;403(10440):2162-203.
4. GBD 2021 Forecasting Collaborators. Burden of disease scenarios for 204 countries and territories, 2022-2050: a forecasting analysis for the Global Burden of Disease Study 2021. Lancet. 2024;403(10440):2204-56.
5. World Health Organization. Handbook on health inequality monitoring: with a special focus on low- and middle-income countries. World Health Organization; 2013. https://www.who.int/publications/i/item/9789241548632.
6. Arnold M, Park JY, Camargo MC, Lunet N, Forman D, Soerjomataram I. Is gastric cancer becoming a rare disease? A global assessment of predicted incidence trends to 2035. Gut. 2020;69(5):823-9.
7. Jiang B, Carriere KC. Age-period-cohort models using smoothing splines: a generalized additive model approach. Stat Med. 2014;33(4):595-606.
8. Chen Q, Li T, Ding H, Huang G, Du D, Yang J. Age-period-cohort analysis of epidemiological trends in pelvic fracture in China from 1992 to 2021 and forecasts for 2046. Front Public Health. 2024;12:1428068.
9. Simões TC, Borges LF, Parreira de Assis AC, Silva MV, Dos Santos J, Meira KC. Chagas disease mortality in Brazil: a Bayesian analysis of age-period-cohort effects and forecasts for two decades. PLoS Negl Trop Dis. 2018;12(9):e0006798.

Tab1e S1 Age-standardized rate (ASR) of disability-adjusted Life years (DALYs) for bladder cancer (BCa) by sex for all countries in 2021

Location	Both sexes			Male			Female		
	Value	95%UI (lower)	95%UI (upper)	Value	95%UI (lower)	95%UI (upper)	Value	95%UI (lower)	95%UI (upper)
Global	51.58	47.56	56.42	86.31	78.87	95.94	23.29	20.99	25.45
Afghanistan	77.68	47.64	105.35	100.47	59.24	148.87	59.46	31.05	96.25
Albania	5.75	4.34	7.80	8.75	5.88	13.07	3.15	2.36	4.12
Algeria	30.51	23.61	38.98	44.76	32.13	59.82	14.29	10.51	19.02
American Samoa	44.00	36.62	53.16	53.14	42.25	65.22	34.99	23.33	43.59
Andorra	74.56	49.57	103.27	146.17	97.42	201.08	4.38	3.13	5.81
Angola	52.74	39.46	69.64	85.18	62.94	116.44	28.58	20.46	39.18
Antigua and Barbuda	52.29	47.88	55.69	61.82	54.00	67.67	44.12	39.93	48.21
Argentina	60.42	55.45	65.23	106.25	96.44	116.40	25.61	22.34	28.86
Armenia	112.35	92.47	132.80	228.57	188.23	270.32	28.56	23.74	33.75
Australia	48.20	43.68	52.69	76.11	68.15	84.14	24.49	20.73	28.19
Austria	62.09	56.44	67.53	106.08	95.14	117.21	28.03	24.60	31.41
Azerbaijan	40.65	28.58	55.46	77.60	54.21	110.95	12.46	8.38	17.65
Bahamas	37.58	30.59	46.23	54.96	44.51	67.62	23.85	19.21	29.17
Bahrain	96.70	70.04	132.26	149.99	105.15	217.70	41.10	31.08	54.20
Bangladesh	22.04	13.45	41.71	33.30	18.85	69.19	9.85	6.77	16.02
Barbados	48.59	38.60	59.62	67.59	52.35	84.14	33.25	26.42	41.01
Belarus	55.72	45.18	67.14	127.70	103.17	153.22	13.65	10.91	17.15
Belgium	79.49	71.84	86.47	133.76	120.34	146.96	35.60	30.37	40.21
Belize	40.84	35.46	46.33	46.70	39.71	54.18	34.68	30.08	38.99
Benin	37.27	28.94	48.66	59.74	44.27	83.02	19.15	13.71	26.56
Bermuda	86.53	73.02	103.77	158.64	133.81	189.32	30.25	24.75	38.98
Bhutan	22.29	14.47	45.69	33.47	20.13	73.69	11.04	7.92	22.87
Bolivia (Plurinational State of)	43.36	31.58	59.85	57.53	41.95	81.24	31.34	20.83	46.52
Bosnia and Herzegovina	85.46	63.25	109.70	149.85	107.19	196.82	35.42	24.84	45.60

Location	Both sexes			Male			Female		
	Value	95%UI (lower)	95%UI (upper)	Value	95%UI (lower)	95%UI (upper)	Value	95%UI (lower)	95%UI (upper)
Botswana	43.92	33.75	59.06	69.01	49.06	99.37	27.32	19.16	39.12
Brazil	47.64	44.07	50.15	71.73	66.33	75.81	29.38	26.55	31.31
Brunei Darussalam	45.95	38.00	56.39	66.44	51.36	84.33	31.88	25.87	38.44
Bulgaria	111.31	93.98	133.06	203.24	170.41	242.91	44.01	36.38	54.16
Burkina Faso	38.62	29.37	53.44	60.12	43.68	85.38	20.25	13.42	28.64
Burundi	50.04	37.23	67.90	61.04	41.38	89.93	38.65	29.39	51.27
Cabo Verde	42.55	21.87	56.76	91.61	46.69	125.81	7.89	2.17	11.27
Cambodia	29.58	21.22	42.14	53.75	36.91	79.81	13.59	9.56	19.85
Cameroon	50.20	38.28	68.37	82.72	60.02	115.36	21.52	15.02	31.22
Canada	60.27	54.52	65.64	95.24	86.65	103.87	31.87	27.38	35.95
Central African Republic	59.74	44.18	78.20	97.22	71.69	130.18	32.89	19.85	48.89
Chad	45.48	33.59	59.33	64.31	45.20	87.11	24.05	18.19	31.82
Chile	47.05	43.24	50.52	72.18	65.77	78.79	27.34	24.52	30.28
China	45.31	36.06	57.41	78.58	59.12	103.70	17.82	13.66	22.57
Colombia	28.42	23.66	33.87	42.49	34.53	51.92	17.22	14.20	19.99
Comoros	45.87	31.50	66.84	52.21	25.00	93.33	40.90	27.82	56.57
Congo	63.36	47.39	83.18	96.17	70.49	132.11	35.02	24.51	46.98
Cook Islands	66.15	52.39	82.97	80.61	61.67	102.18	52.51	39.16	70.64
Costa Rica	39.11	34.11	43.92	62.28	53.61	71.05	19.79	17.28	22.38
Coted'Ivoire	49.17	37.22	68.83	59.34	42.55	85.40	38.67	28.94	52.19
Croatia	106.96	91.19	124.53	185.34	155.20	221.46	49.92	42.04	58.54
Cuba	79.23	67.80	89.95	128.66	108.57	148.46	36.81	31.07	42.93
Cyprus	77.19	60.52	98.45	138.89	103.68	186.03	27.19	22.46	33.32
Czechia	97.06	80.87	115.62	162.11	132.69	193.74	48.18	40.19	57.33
Democratic People's Republic of Korea	40.83	30.49	59.89	71.41	52.24	105.64	20.08	14.42	31.31
Democratic Republic of the Congo	54.13	39.45	72.26	84.42	55.33	126.94	32.42	23.04	42.92
Denmark	87.49	79.88	95.14	135.34	122.64	147.92	46.72	40.42	53.16
Djibouti	56.04	38.31	81.61	66.90	38.44	109.68	44.59	29.52	68.75

Location	Both sexes			Male			Female		
	Value	95%UI (lower)	95%UI (upper)	Value	95%UI (lower)	95%UI (upper)	Value	95%UI (lower)	95%UI (upper)
Dominica	68.35	54.73	83.96	88.83	64.00	120.32	51.47	42.55	61.04
Dominican Republic	23.25	16.97	31.72	33.43	21.82	48.67	13.99	11.06	17.63
Ecuador	28.44	22.38	36.28	38.25	29.11	49.39	20.17	15.66	25.42
Egypt	91.71	65.83	153.70	142.23	99.63	249.84	32.28	25.62	52.61
El Salvador	20.10	16.03	24.70	25.04	18.09	32.98	16.54	13.35	20.44
Equatorial Guinea	57.92	40.37	80.64	98.15	65.25	139.83	29.45	18.72	43.22
Eritrea	60.82	45.48	81.10	77.19	56.21	108.08	50.95	34.66	69.94
Estonia	73.32	63.32	84.19	151.32	130.27	174.47	27.42	22.42	33.30
Eswatini	63.62	43.47	87.26	109.22	71.68	151.24	36.30	20.96	56.28
Ethiopia	48.41	34.06	68.92	52.94	31.45	88.02	43.73	32.37	59.41
Fiji	38.46	23.76	51.12	57.44	32.75	77.32	24.61	13.31	32.72
Finland	43.77	39.94	47.90	72.21	65.37	79.40	20.78	17.53	23.35
France	89.01	79.53	99.78	161.83	142.67	182.31	31.80	26.86	36.07
Gabon	69.40	51.73	93.20	117.36	87.35	164.26	31.28	20.59	43.02
Gambia	29.62	21.37	38.31	42.23	28.37	58.25	18.76	14.14	23.88
Georgia	115.52	98.94	132.03	240.40	204.52	276.11	31.04	26.86	35.77
Germany	67.88	62.05	73.35	111.38	101.74	121.02	33.27	28.64	36.66
Ghana	50.21	37.49	66.16	88.49	62.79	126.95	21.90	16.13	30.44
Greece	116.05	107.00	124.78	212.82	196.42	229.24	34.81	30.81	38.02
Greenland	70.95	49.77	91.11	110.24	71.80	145.80	30.18	23.70	38.11
Grenada	56.66	47.74	64.02	88.39	75.17	102.05	35.68	30.49	41.29
Guam	32.25	27.00	37.84	56.03	45.90	66.62	10.19	8.15	12.06
Guatemala	15.87	13.50	18.27	19.17	16.14	22.35	13.07	11.38	14.95
Guinea	70.01	52.28	95.40	113.38	79.78	158.57	25.76	18.65	35.77
Guinea-Bissau	55.89	41.38	72.96	91.53	64.66	126.06	27.80	19.71	36.78
Guyana	36.43	27.52	46.87	53.68	39.57	70.21	21.73	16.60	28.30
Haiti	60.14	42.47	81.33	79.33	51.77	116.39	42.59	24.35	72.19
Honduras	14.53	10.87	19.32	19.75	12.18	28.98	10.05	7.81	13.00

Location	Both sexes			Male			Female		
	Value	95%UI (lower)	95%UI (upper)	Value	95%UI (lower)	95%UI (upper)	Value	95%UI (lower)	95%UI (upper)
Hungary	117.44	102.29	135.01	201.22	173.15	233.01	58.47	49.11	68.68
Iceland	60.44	53.37	68.06	90.96	80.12	103.38	33.08	27.82	38.51
India	24.94	21.66	29.37	40.33	33.58	49.26	11.20	9.80	12.88
Indonesia	24.60	16.69	40.60	39.27	24.33	72.17	11.64	7.85	16.75
Iran (Islamic Republic of)	45.60	37.59	52.04	72.30	56.79	85.12	19.10	16.80	21.68
Iraq	110.08	79.35	146.45	171.61	112.88	245.82	55.24	43.07	70.03
Ireland	55.66	49.02	61.90	84.18	73.47	94.65	31.15	25.97	37.47
Israel	69.57	61.99	76.14	123.01	109.80	135.54	25.40	21.41	28.62
Italy	92.64	84.88	100.09	168.60	155.06	181.43	33.83	29.38	37.45
Jamaica	51.24	38.84	66.22	65.74	49.02	85.40	37.98	29.07	49.75
Japan	40.57	36.57	43.41	66.98	61.88	70.96	19.54	16.20	21.60
Jordan	53.03	38.31	72.54	85.36	59.66	118.90	17.44	12.18	24.94
Kazakhstan	45.13	37.89	53.36	94.97	79.14	113.72	13.99	11.72	16.43
Kenya	25.31	20.43	30.43	30.97	22.96	39.18	21.00	15.55	28.50
Kiribati	13.40	9.74	16.82	11.09	7.98	14.84	15.04	9.16	19.59
Kuwait	59.47	48.74	72.94	84.73	68.12	105.76	23.96	18.71	28.91
Kyrgyzstan	32.85	26.33	40.45	63.59	50.31	78.63	10.86	8.88	13.13
Lao People's Democratic Republic	27.33	19.16	40.65	44.23	28.14	67.23	12.34	8.23	18.46
Latvia	98.33	83.69	114.57	214.82	180.21	253.63	30.05	24.77	35.93
Lebanon	147.63	116.80	186.45	252.37	183.93	339.95	59.05	43.90	76.64
Lesotho	54.10	34.51	76.28	86.71	49.74	131.99	35.97	21.82	53.85
Liberia	41.93	30.22	59.19	59.37	41.08	90.07	23.57	16.65	32.83
Libya	127.33	91.69	174.49	211.77	146.48	300.58	42.98	31.91	55.37
Lithuania	83.56	72.32	94.00	180.05	155.83	204.16	27.41	22.88	31.91
Luxembourg	71.97	64.30	80.56	117.14	104.76	131.75	35.14	30.64	40.00
Madagascar	41.36	30.36	52.33	46.71	31.58	66.24	36.76	25.67	49.16
Malawi	179.92	140.23	227.23	230.55	166.00	311.16	142.83	99.03	208.26
Malaysia	52.10	38.15	64.95	81.20	53.94	106.08	23.65	18.56	27.84

Location	Both sexes			Male			Female		
	Value	95%UI (lower)	95%UI (upper)	Value	95%UI (lower)	95%UI (upper)	Value	95%UI (lower)	95%UI (upper)
Maldives	19.40	14.75	24.76	29.50	21.66	38.79	7.79	6.16	9.68
Mali	176.81	134.33	231.64	249.42	175.10	344.94	99.33	74.13	138.98
Malta	74.12	65.36	83.04	117.97	103.62	134.19	36.78	31.43	42.22
Marshall Islands	46.51	31.61	63.08	69.61	46.10	100.92	22.64	11.98	34.13
Mauritania	38.47	25.84	55.18	53.74	30.54	84.68	23.02	16.30	31.72
Mauritius	47.69	44.07	50.57	79.61	73.79	85.25	21.73	19.55	23.47
Mexico	28.42	24.92	32.28	42.10	35.25	50.02	16.68	14.26	19.16
Micronesia (Federated States of)	45.68	31.33	62.27	70.68	45.34	100.36	24.33	13.83	32.74
Monaco	105.29	49.74	238.31	163.39	73.59	482.53	56.40	20.73	139.05
Mongolia	28.31	21.53	36.61	44.53	31.09	63.05	16.80	11.53	25.04
Montenegro	91.67	71.77	117.80	158.17	114.04	214.87	41.51	34.01	49.88
Morocco	28.46	19.80	36.99	51.47	35.29	69.45	6.46	4.58	8.66
Mozambique	63.82	47.23	85.52	87.42	65.06	122.09	47.12	32.12	68.11
Myanmar	24.45	17.43	37.65	41.07	28.72	66.29	12.44	9.29	19.98
Namibia	35.76	27.69	45.91	51.80	40.68	67.33	25.07	17.09	34.92
Nauru	56.22	36.90	79.87	90.56	54.64	136.19	28.99	15.88	42.35
Nepal	23.83	15.96	47.54	37.06	23.22	78.71	12.12	8.76	22.67
Netherlands	75.99	69.22	82.31	126.61	113.78	137.60	35.09	30.35	40.02
New Zealand	56.90	51.07	62.42	92.43	82.64	103.39	26.76	22.50	31.02
Nicaragua	12.78	10.43	15.80	16.48	12.35	21.71	9.89	7.86	12.46
Niger	28.58	19.92	45.29	39.18	23.83	69.56	18.57	10.61	26.57
Nigeria	14.16	11.35	18.59	16.86	11.56	28.24	11.77	8.51	16.21
Niue	39.85	28.50	50.87	65.83	45.38	86.56	18.81	10.16	25.10
North Macedonia	100.88	77.58	129.76	175.76	128.45	230.78	37.36	29.26	47.99
Northern Mariana Islands	71.84	54.60	81.43	75.52	61.03	90.17	69.53	33.73	82.43
Norway	66.71	60.60	71.44	107.73	98.04	115.74	32.39	28.73	35.67
Oman	30.01	23.38	37.88	44.20	32.02	59.01	17.06	13.13	21.24
Pakistan	103.37	81.05	136.35	145.93	102.77	205.14	56.47	42.76	74.98

Location	Both sexes			Male			Female		
	Value	95%UI (lower)	95%UI (upper)	Value	95%UI (lower)	95%UI (upper)	Value	95%UI (lower)	95%UI (upper)
Palau	14.10	10.28	18.49	25.15	18.11	33.12	1.65	1.31	2.08
Palestine	66.77	53.47	83.71	123.53	96.04	162.22	20.34	16.83	24.18
Panama	22.77	17.83	27.46	30.54	23.29	37.90	15.71	12.60	18.73
Papua New Guinea	29.18	17.18	44.55	38.05	20.19	62.57	19.02	10.26	25.68
Paraguay	28.73	20.71	37.77	47.54	32.34	65.63	12.78	9.53	16.31
Peru	26.24	19.23	35.04	34.95	23.59	49.71	18.41	14.09	23.54
Philippines	20.62	16.83	27.66	33.21	25.22	48.20	10.30	8.28	12.84
Poland	136.94	123.78	150.32	255.26	227.52	283.96	55.13	49.19	60.82
Portugal	81.97	74.80	90.46	147.79	134.10	163.56	31.07	27.00	35.09
Puerto Rico	44.54	36.52	52.85	68.88	56.20	82.34	24.78	20.23	29.83
Qatar	63.49	44.64	93.27	83.76	53.29	126.46	35.47	25.40	46.37
Republic of Korea	37.18	28.39	45.45	70.11	49.70	88.90	14.30	10.73	17.10
Republic of Moldova	70.62	62.95	79.04	145.40	128.28	162.62	19.34	16.42	22.70
Romania	105.52	93.02	119.11	195.14	170.70	222.75	38.02	30.62	46.69
Russian Federation	62.39	56.50	68.67	132.55	117.61	147.05	21.09	18.89	23.18
Rwanda	49.69	33.55	75.07	63.26	36.21	105.46	41.05	22.57	61.00
Saint Kitts and Nevis	59.83	49.91	68.44	77.50	62.29	90.45	46.23	38.65	53.90
Saint Lucia	60.31	49.19	71.91	81.94	65.52	99.23	42.24	34.81	50.47
Saint Vincent and the Grenadines	51.89	45.77	58.98	78.73	68.59	90.56	25.80	22.24	29.81
Samoa	25.20	18.03	36.03	51.25	36.67	72.76	1.00	0.77	1.24
San Marino	72.93	46.70	106.37	122.52	77.50	180.92	28.05	18.15	39.77
Sao Tome and Principe	88.61	64.65	117.07	156.21	106.61	217.57	30.53	24.13	39.41
Saudi Arabia	35.73	25.01	59.71	47.88	30.63	84.96	17.59	13.10	24.69
Senegal	45.75	34.40	61.14	70.78	50.16	99.01	23.52	17.00	32.03
Serbia	102.12	76.65	130.08	175.90	123.99	235.04	43.25	34.30	52.50
Seychelles	76.38	63.77	95.63	115.18	91.02	149.85	43.22	36.36	51.02
Sierra Leone	39.12	29.24	52.75	57.65	42.12	83.56	20.97	15.60	27.43
Singapore	25.70	22.99	28.28	40.21	35.84	44.35	13.60	11.32	15.54

Location	Both sexes			Male			Female		
	Value	95%UI (lower)	95%UI (upper)	Value	95%UI (lower)	95%UI (upper)	Value	95%UI (lower)	95%UI (upper)
Slovakia	84.70	66.73	110.09	155.11	114.31	210.57	34.89	26.89	43.73
Slovenia	85.82	73.01	99.13	145.77	125.41	168.74	40.45	33.42	48.42
Solomon Islands	39.84	24.28	58.07	57.37	32.47	90.69	22.09	12.24	31.76
Somalia	55.37	39.46	73.30	63.06	34.74	102.11	50.50	31.31	75.26
South Africa	49.85	43.33	56.80	83.87	69.82	99.31	28.08	24.67	31.89
South Sudan	53.01	37.49	72.62	61.71	41.82	93.62	43.21	28.58	61.51
Spain	99.12	90.21	108.64	188.10	170.00	207.20	28.93	24.86	32.72
Sri Lanka	19.15	12.23	27.58	34.67	21.40	50.32	7.44	4.94	10.36
Sudan	55.87	41.33	75.03	80.25	57.96	111.26	26.48	18.86	38.46
Suriname	33.54	24.45	44.82	53.50	36.13	77.36	17.10	13.26	21.16
Sweden	59.81	52.57	67.19	90.73	77.83	104.15	33.40	27.95	38.77
Switzerland	56.56	49.97	63.65	91.32	80.88	103.73	27.92	23.59	31.86
Syrian Arab Republic	49.89	35.54	68.74	81.54	55.13	115.16	15.08	11.10	20.15
Taiwan (Province of China)	62.79	55.74	69.40	95.18	83.09	106.19	34.94	29.93	39.02
Tajikistan	16.37	12.12	21.68	27.02	18.18	37.18	6.33	4.64	8.51
Thailand	43.23	32.13	56.59	74.67	53.85	99.62	17.05	12.83	22.37
Timor-Leste	20.77	14.92	30.51	30.33	19.94	46.90	11.47	8.29	17.54
Togo	44.56	33.66	58.93	76.90	54.17	106.29	22.56	16.39	31.75
Tokelau	35.00	23.79	47.97	51.77	34.86	74.15	19.69	10.40	25.88
Tonga	31.92	20.15	49.00	51.16	31.35	83.47	15.47	8.38	22.45
Trinidad and Tobago	38.93	30.25	49.36	57.23	43.43	72.47	22.69	17.40	28.52
Tunisia	69.15	46.83	97.07	122.79	79.73	176.12	21.15	15.00	29.23
Turkey	84.07	64.53	106.59	158.09	119.04	204.56	21.31	17.08	26.81
Turkmenistan	28.55	21.99	37.01	52.22	40.14	67.56	10.51	8.05	13.92
Tuvalu	39.44	27.36	50.12	59.76	38.74	80.51	21.96	12.34	29.80
Uganda	59.95	45.06	77.75	83.17	60.61	113.70	43.58	32.51	60.81
Ukraine	66.20	46.52	89.23	143.04	96.84	199.07	18.40	11.88	26.94
United Arab Emirates	87.21	67.29	110.35	86.87	64.53	114.91	91.42	71.98	114.64

Location	Both sexes			Male			Female		
	Value	95%UI (lower)	95%UI (upper)	Value	95%UI (lower)	95%UI (upper)	Value	95%UI (lower)	95%UI (upper)
United Kingdom	80.78	75.02	84.53	120.59	112.82	125.85	48.07	43.67	50.80
United Republic of Tanzania	48.43	35.72	66.07	56.44	36.62	85.76	41.56	30.61	57.65
United States of America	68.15	62.69	72.49	111.05	102.51	117.79	33.73	30.22	36.13
United States Virgin Islands	23.21	17.26	30.10	41.61	28.49	56.42	9.20	6.20	13.90
Uruguay	100.18	91.68	109.47	185.44	167.68	205.16	38.97	34.97	43.27
Uzbekistan	27.38	21.26	35.10	48.02	36.89	61.58	11.13	8.63	14.22
Vanuatu	37.37	25.16	50.26	57.38	35.03	85.09	18.39	10.17	26.16
Venezuela (Bolivarian Republic of)	37.85	29.00	48.37	53.71	40.49	70.46	25.03	18.83	32.15
Viet Nam	24.51	18.86	30.02	49.32	37.88	61.44	7.36	5.55	9.97
Yemen	61.25	43.31	82.60	94.18	64.12	134.63	29.93	21.24	42.43
Zambia	74.68	45.20	130.34	94.59	51.55	185.23	57.54	31.09	108.97
Zimbabwe	169.21	121.98	220.28	212.31	165.88	272.81	139.87	85.09	209.22

UI uncertainty interval

Table S2 Age-standardized rate (ASR) of disability-adjusted Life years (DALYs) for kidney cancer (KCa) by sex for all countries in 2021

Location	Both sexes			Male			Female		
	Value	95%UI (lower)	95%UI (upper)	Value	95%UI (lower)	95%UI (upper)	Value	95%UI (lower)	95%UI (upper)
Global	47.33	44.76	50.07	67.79	63.83	72.46	28.91	26.54	31.21
Afghanistan	34.54	23.19	55.72	34.74	24.75	48.01	34.68	18.39	76.96
Albania	52.01	39.77	67.09	78.96	58.76	104.04	27.46	18.82	38.62
Algeria	13.35	10.77	16.39	17.15	13.12	21.54	9.39	6.53	12.54
American Samoa	24.12	18.75	30.64	42.30	32.73	54.12	5.56	4.04	7.48
Andorra	55.10	36.57	76.59	75.49	48.11	106.44	33.80	22.85	46.93
Angola	23.01	14.27	34.75	39.50	21.74	61.27	9.71	4.80	15.77
Antigua and Barbuda	47.08	44.03	50.36	59.57	54.56	64.68	35.68	33.01	38.68
Argentina	125.45	114.18	137.27	192.08	171.48	216.09	69.14	60.31	79.39
Armenia	72.93	56.20	92.01	108.19	83.54	136.13	45.68	35.10	58.34
Australia	66.37	58.68	74.88	100.05	88.84	112.58	35.53	30.47	40.66
Austria	69.19	63.40	74.31	95.10	87.24	102.51	47.18	42.26	51.25
Azerbaijan	85.44	57.46	121.69	130.44	80.51	199.34	46.87	28.09	73.08
Bahamas	65.73	52.07	83.27	86.54	67.54	109.44	47.82	38.31	59.63
Bahrain	57.26	45.76	69.46	73.91	57.74	91.57	36.79	28.22	46.52
Bangladesh	16.14	11.45	22.18	23.31	15.40	33.02	8.69	6.29	12.36
Barbados	70.22	54.12	89.05	92.75	70.09	118.94	51.01	39.78	64.77
Belarus	109.87	84.43	138.84	187.39	145.36	235.17	54.70	42.16	69.51
Belgium	68.95	60.92	77.21	97.55	84.92	111.29	43.76	36.91	51.09
Belize	49.11	42.84	55.86	57.63	48.45	66.48	40.37	35.84	45.85
Benin	22.88	17.41	29.05	33.76	26.05	43.73	12.66	7.55	18.09
Bermuda	59.43	49.23	71.55	92.44	76.05	111.74	30.53	24.70	38.53
Bhutan	21.39	14.56	29.88	27.81	17.74	40.99	14.67	9.43	21.11
Bolivia (Plurinational State of)	66.62	49.86	90.03	75.27	54.37	102.35	58.59	40.30	84.81
Bosnia and Herzegovina	87.75	67.73	108.51	127.05	94.63	163.49	54.05	41.07	69.47
Botswana	36.35	27.34	47.57	51.60	33.81	73.17	24.57	15.52	34.64

Location	Both sexes			Male			Female		
	Value	95%UI (lower)	95%UI (upper)	Value	95%UI (lower)	95%UI (upper)	Value	95%UI (lower)	95%UI (upper)
Brazil	54.12	51.12	56.79	74.05	69.08	78.50	37.47	34.74	39.82
Brunei Darussalam	65.14	55.09	76.66	84.23	69.76	100.44	48.29	38.46	60.07
Bulgaria	83.10	65.45	103.41	129.60	103.14	160.98	43.71	33.78	55.51
Burkina Faso	20.27	15.35	25.32	29.07	21.92	36.66	11.97	7.08	17.04
Burundi	28.40	14.52	43.13	36.78	17.90	55.47	19.20	10.05	32.61
Cabo Verde	43.93	32.44	56.31	72.49	50.90	98.06	19.14	14.22	25.14
Cambodia	29.17	22.16	37.44	43.67	32.49	56.88	18.43	13.40	25.61
Cameroon	27.00	19.14	37.40	40.22	25.76	61.70	14.08	7.40	20.98
Canada	68.79	60.11	78.24	101.04	88.11	115.67	39.72	34.43	45.21
Central African Republic	19.54	12.81	29.73	33.24	21.55	52.79	8.44	4.95	14.69
Chad	18.42	13.64	24.82	25.55	18.02	36.06	10.40	7.24	15.59
Chile	100.09	90.51	110.03	149.74	134.43	165.52	57.31	50.93	63.24
China	34.18	28.29	40.77	50.81	40.15	63.77	18.46	13.59	23.84
Colombia	39.93	32.60	47.56	52.92	43.01	64.24	29.00	24.03	34.37
Comoros	41.46	26.13	57.40	50.81	27.08	74.71	33.25	21.30	48.14
Congo	29.85	21.22	40.19	45.64	30.43	63.68	14.96	9.27	21.83
Cook Islands	6.31	4.98	8.04	11.54	9.02	14.77	1.40	0.95	2.03
Costa Rica	68.60	58.16	78.03	92.18	77.50	106.67	48.21	41.03	54.34
Coted'Ivoire	10.84	6.77	15.28	15.15	9.28	21.99	6.09	3.67	9.57
Croatia	97.91	76.56	124.94	147.82	115.11	190.79	56.57	45.24	69.97
Cuba	50.60	42.80	58.92	70.71	58.73	83.40	32.32	27.14	37.80
Cyprus	41.14	32.26	49.92	63.79	47.95	80.60	21.11	15.97	27.27
Czechia	139.52	116.42	165.61	202.60	165.92	243.37	86.03	72.62	100.57
Democratic People’s Republic of Korea	29.18	20.56	39.30	42.86	31.76	57.62	17.82	9.34	26.77
Democratic Republic of the Congo	18.35	9.79	30.82	31.49	15.82	53.82	7.40	4.02	13.22
Denmark	72.81	64.20	82.03	101.69	89.39	114.75	46.15	39.68	52.78
Djibouti	40.80	22.28	63.18	51.85	23.50	84.79	27.77	13.76	44.42
Dominica	66.89	53.86	83.02	90.95	70.83	116.26	43.50	34.07	54.81

Location	Both sexes			Male			Female		
	Value	95%UI (lower)	95%UI (upper)	Value	95%UI (lower)	95%UI (upper)	Value	95%UI (lower)	95%UI (upper)
Dominican Republic	34.56	25.74	47.04	43.48	29.12	65.88	25.90	20.28	33.73
Ecuador	45.56	35.62	58.12	56.77	43.45	74.00	35.27	27.87	43.73
Egypt	23.48	19.33	28.62	28.52	22.93	34.77	18.10	14.38	22.43
El Salvador	39.62	32.01	48.66	51.29	40.20	65.06	31.10	24.12	38.64
Equatorial Guinea	31.85	21.09	44.14	54.57	35.64	77.21	14.43	6.69	25.39
Eritrea	33.71	18.24	51.94	41.93	18.49	67.24	27.33	12.92	42.48
Estonia	112.28	95.56	130.30	175.98	148.42	206.10	66.73	55.36	77.61
Eswatini	51.07	32.91	82.34	76.14	41.69	143.01	33.20	20.92	49.26
Ethiopia	38.57	26.04	52.19	43.85	26.79	61.17	33.15	22.59	46.93
Fiji	9.37	6.89	12.08	14.03	10.26	18.77	5.46	4.01	7.17
Finland	78.11	69.64	86.38	104.35	92.54	115.94	54.34	47.35	61.38
France	83.36	74.19	92.64	126.28	111.06	141.64	46.73	40.74	53.24
Gabon	36.82	26.71	48.68	58.57	44.15	77.26	17.39	10.24	26.08
Gambia	13.27	9.65	17.76	15.25	10.71	20.37	11.32	7.08	18.16
Georgia	87.31	68.79	108.24	151.47	119.22	187.95	37.56	29.86	47.27
Germany	80.55	71.81	89.87	119.48	106.47	132.46	46.38	39.81	53.63
Ghana	22.48	16.99	28.39	32.20	24.54	41.46	14.10	9.42	19.74
Greece	74.49	68.17	80.72	112.01	101.74	121.52	41.30	37.86	45.07
Greenland	131.29	104.49	163.29	175.40	135.36	231.18	81.43	61.75	102.79
Grenada	59.69	51.23	68.87	86.08	73.27	99.98	36.15	31.35	41.02
Guam	37.62	31.78	43.93	71.96	60.69	84.51	4.46	3.75	5.35
Guatemala	39.83	33.90	46.65	45.57	37.92	54.29	34.88	29.87	40.60
Guinea	19.62	11.94	28.69	31.72	19.37	46.26	7.21	4.18	13.45
Guinea-Bissau	19.85	15.36	24.81	29.40	21.21	37.56	11.36	7.81	15.87
Guyana	55.79	42.09	71.63	68.46	49.63	88.20	44.30	33.48	55.78
Haiti	41.68	27.58	64.71	49.79	30.35	76.98	34.41	19.68	71.89
Honduras	26.95	21.34	34.04	30.65	22.90	41.90	23.64	17.31	31.62
Hungary	113.58	94.86	137.61	165.37	137.21	199.96	71.51	59.83	86.08

Location	Both sexes			Male			Female		
	Value	95%UI (lower)	95%UI (upper)	Value	95%UI (lower)	95%UI (upper)	Value	95%UI (lower)	95%UI (upper)
Iceland	108.51	95.13	123.65	139.36	121.45	159.20	78.79	68.36	90.30
India	18.40	16.48	20.49	26.09	22.64	29.93	11.06	9.44	12.81
Indonesia	28.87	23.68	35.64	38.35	29.15	49.75	19.91	13.72	27.42
Iran (Islamic Republic of)	30.71	28.19	33.18	40.38	35.75	45.63	21.05	17.85	24.22
Iraq	43.46	31.94	54.61	64.63	46.65	83.37	22.65	16.57	29.31
Ireland	66.63	58.12	75.29	95.31	82.06	108.06	40.14	34.31	46.28
Israel	56.64	49.92	63.26	84.79	73.83	95.18	31.87	27.77	35.92
Italy	72.40	66.42	77.19	107.89	100.11	114.63	41.86	37.39	45.53
Jamaica	43.70	32.46	56.34	53.55	38.32	70.67	34.37	25.52	44.97
Japan	42.54	39.44	44.58	64.39	60.89	67.06	23.33	20.60	25.06
Jordan	30.41	23.63	38.73	44.60	33.92	57.60	14.38	10.09	20.66
Kazakhstan	73.09	61.17	84.13	113.08	94.67	132.62	43.05	35.58	50.24
Kenya	19.92	14.76	25.54	20.60	14.27	26.40	19.25	13.85	27.02
Kiribati	38.69	26.68	56.40	86.11	60.05	124.67	1.20	0.79	2.00
Kuwait	34.81	27.53	42.94	47.39	36.59	60.00	17.62	14.54	21.01
Kyrgyzstan	61.35	49.54	76.27	86.35	68.76	108.08	41.66	33.12	51.62
Lao People’s Democratic Republic	27.51	19.98	37.35	36.13	26.11	48.81	19.44	12.44	28.31
Latvia	135.11	111.33	163.08	210.07	174.57	254.96	82.06	66.55	98.38
Lebanon	41.56	33.05	51.64	70.92	54.55	90.21	16.41	11.90	23.92
Lesotho	39.01	28.88	52.62	58.76	40.62	89.61	26.28	15.74	40.95
Liberia	20.51	14.93	27.90	30.59	20.46	44.91	9.75	3.64	16.24
Libya	71.11	51.41	97.83	110.51	76.42	157.71	30.94	20.20	43.79
Lithuania	135.35	114.08	157.18	223.93	189.50	263.91	72.16	59.87	84.44
Luxembourg	39.50	35.14	44.28	55.07	48.60	61.77	25.17	22.36	28.05
Madagascar	26.20	15.03	39.55	31.74	13.94	50.95	20.94	11.28	30.55
Malawi	69.39	43.53	97.08	98.67	61.08	138.37	44.04	25.74	75.11
Malaysia	34.76	30.04	41.00	48.70	41.06	58.88	20.83	16.33	25.81
Maldives	13.50	10.57	16.99	21.29	16.29	27.17	4.04	3.20	5.20

Location	Both sexes			Male			Female		
	Value	95%UI (lower)	95%UI (upper)	Value	95%UI (lower)	95%UI (upper)	Value	95%UI (lower)	95%UI (upper)
Mali	22.23	14.83	31.62	27.66	17.53	39.55	16.52	10.83	26.87
Malta	69.71	60.25	79.85	94.12	81.00	107.75	46.79	40.09	54.60
Marshall Islands	15.30	9.59	24.51	27.13	16.72	45.22	3.13	2.00	6.91
Mauritania	22.76	14.74	31.40	32.84	20.96	46.82	12.65	8.07	18.48
Mauritius	27.48	25.09	29.31	43.35	39.48	46.56	13.37	12.14	14.33
Mexico	83.65	73.55	94.66	114.03	94.03	135.74	56.86	48.39	66.22
Micronesia (Federated States of)	16.93	11.78	23.04	31.77	22.52	43.45	3.47	1.92	5.06
Monaco	111.24	89.23	137.28	138.50	109.94	175.67	86.31	64.86	111.48
Mongolia	73.17	58.91	89.63	94.25	72.26	122.09	57.10	42.03	73.80
Montenegro	85.71	71.75	104.10	131.37	106.56	165.09	46.79	35.43	59.12
Morocco	7.10	5.40	8.71	9.36	6.92	11.92	4.85	3.43	6.63
Mozambique	19.92	14.77	25.96	27.57	21.08	36.01	13.61	8.93	20.05
Myanmar	25.75	20.34	33.28	33.29	24.61	43.66	19.93	14.03	26.65
Namibia	48.01	36.05	63.35	64.38	49.65	84.11	35.34	23.82	50.25
Nauru	19.79	14.10	27.57	36.14	24.78	51.71	6.11	4.30	8.47
Nepal	16.92	12.33	22.93	24.65	16.50	37.63	10.02	6.32	14.36
Netherlands	84.04	74.87	94.25	119.48	106.46	134.26	51.67	44.96	59.33
New Zealand	74.01	65.01	83.34	106.95	94.82	119.64	43.70	37.65	50.27
Nicaragua	34.13	27.69	42.30	44.82	34.93	58.41	25.14	19.41	31.12
Niger	14.59	8.60	21.41	20.88	12.16	30.41	8.52	4.88	13.24
Nigeria	29.03	20.43	37.96	41.97	28.86	57.05	17.37	12.41	23.69
Niue	24.48	19.23	31.00	40.28	30.95	52.65	10.68	7.01	15.93
North Macedonia	45.66	36.04	55.76	67.52	52.07	86.05	25.27	19.23	32.45
Northern Mariana Islands	41.68	34.25	48.88	78.51	64.49	92.16	3.58	2.94	4.32
Norway	67.44	62.01	72.53	95.71	88.25	102.54	40.65	36.70	44.48
Oman	20.63	16.14	26.45	30.03	22.84	40.82	9.40	6.61	12.71
Pakistan	28.47	23.38	35.21	40.33	31.25	52.38	15.56	11.59	21.45
Palau	8.71	6.54	11.52	15.06	11.18	20.16	1.33	1.02	1.69

Location	Both sexes			Male			Female		
	Value	95%UI (lower)	95%UI (upper)	Value	95%UI (lower)	95%UI (upper)	Value	95%UI (lower)	95%UI (upper)
Palestine	33.20	28.15	39.90	48.15	39.33	60.61	19.62	15.75	24.35
Panama	64.68	49.77	79.31	78.75	58.59	98.48	51.33	41.58	61.50
Papua New Guinea	9.92	4.68	16.79	16.04	6.34	28.81	3.04	1.91	5.04
Paraguay	54.64	40.65	71.47	72.14	51.44	99.73	38.56	28.83	51.13
Peru	57.11	42.39	75.72	73.31	53.31	100.02	41.92	31.19	55.83
Philippines	34.53	29.57	40.08	46.96	37.49	56.71	23.25	18.89	28.60
Poland	113.08	101.94	123.65	170.26	151.31	189.61	67.40	59.06	75.74
Portugal	47.29	41.66	53.85	73.03	63.58	83.28	25.99	22.45	29.60
Puerto Rico	55.71	45.37	65.97	82.30	65.28	99.47	32.83	26.62	39.46
Qatar	45.16	31.64	61.51	56.86	38.41	81.40	25.49	18.87	33.96
Republic of Korea	36.79	31.83	41.36	58.58	49.47	67.68	18.17	14.31	21.99
Republic of Moldova	67.19	59.46	76.63	113.78	100.50	129.87	31.60	27.23	36.15
Romania	83.76	69.33	97.53	121.49	99.96	141.40	51.29	42.53	60.03
Russian Federation	105.88	97.01	115.12	170.05	150.44	187.95	60.87	54.25	67.36
Rwanda	38.59	22.76	54.27	49.39	26.08	76.65	30.34	19.70	46.48
Saint Kitts and Nevis	69.14	57.04	81.56	93.79	76.73	110.82	47.05	38.70	56.39
Saint Lucia	50.42	40.61	60.95	69.46	55.43	84.79	32.51	26.08	39.51
Saint Vincent and the Grenadines	52.23	45.45	59.56	48.69	41.57	55.75	55.89	48.29	64.32
Samoa	10.44	8.26	13.67	13.17	9.75	17.78	7.71	5.54	10.58
San Marino	31.74	19.54	46.08	48.37	29.77	72.49	16.65	10.07	24.47
Sao Tome and Principe	16.79	10.75	27.32	32.48	20.92	52.99	1.51	1.05	2.16
Saudi Arabia	38.08	29.66	47.62	53.06	40.14	68.31	15.30	11.06	19.99
Senegal	21.19	13.47	29.65	31.42	18.75	45.91	11.40	7.58	16.91
Serbia	85.08	67.14	102.46	124.81	94.49	159.28	50.16	39.19	61.66
Seychelles	34.20	29.29	39.22	48.96	41.18	57.58	19.87	16.22	24.80
Sierra Leone	18.62	12.74	26.31	26.74	18.28	38.52	10.21	5.85	15.17
Singapore	36.24	32.18	40.61	50.18	44.59	56.29	22.95	19.61	26.19
Slovakia	129.42	106.95	153.73	197.62	157.56	245.94	73.18	54.64	93.46

Location	Both sexes			Male			Female		
	Value	95%UI (lower)	95%UI (upper)	Value	95%UI (lower)	95%UI (upper)	Value	95%UI (lower)	95%UI (upper)
Slovenia	82.20	68.81	99.93	121.24	101.92	145.81	48.34	38.06	60.75
Solomon Islands	13.45	7.85	19.25	24.02	13.67	34.99	2.72	1.66	4.04
Somalia	23.97	14.45	34.73	36.73	23.98	50.68	14.46	5.27	25.00
South Africa	39.98	36.25	43.30	60.29	53.86	66.98	24.86	21.81	28.35
South Sudan	44.22	32.21	58.87	50.10	36.38	70.96	37.78	23.87	57.72
Spain	68.27	58.89	78.19	105.23	90.53	120.84	36.04	30.37	41.41
Sri Lanka	21.21	14.01	29.00	32.57	20.38	46.75	11.83	7.59	16.34
Sudan	32.36	23.21	45.13	42.01	27.81	62.80	21.19	10.87	34.11
Suriname	44.34	33.98	56.88	57.24	42.22	76.10	32.78	25.05	42.56
Sweden	60.75	52.59	69.78	78.37	65.56	92.14	44.25	37.93	51.52
Switzerland	45.28	40.00	50.91	64.22	56.50	72.65	28.25	24.30	32.81
Syrian Arab Republic	32.12	22.80	44.88	45.98	28.82	72.53	17.50	7.06	27.71
Taiwan (Province of China)	69.05	62.00	76.36	91.11	80.15	102.38	48.91	43.50	54.27
Tajikistan	41.53	30.39	54.25	60.84	40.97	86.36	22.90	16.36	31.77
Thailand	33.41	25.75	42.85	51.76	38.87	68.54	17.07	13.10	21.62
Timor-Leste	19.97	14.29	26.91	26.42	17.43	36.68	13.47	9.98	18.65
Togo	18.59	11.48	27.31	29.11	19.06	43.56	9.72	4.92	15.20
Tokelau	27.04	13.05	39.77	44.69	19.57	70.98	9.35	5.70	17.56
Tonga	15.90	10.36	21.33	30.30	19.98	41.00	3.02	1.73	4.79
Trinidad and Tobago	59.80	45.39	76.16	70.03	52.67	90.01	50.17	38.12	64.18
Tunisia	32.50	22.87	45.37	49.45	33.62	70.35	16.52	11.80	23.87
Turkey	51.88	42.20	63.57	76.20	60.51	96.75	29.58	23.25	36.55
Turkmenistan	99.44	75.42	130.00	156.12	118.17	203.99	51.73	39.20	67.76
Tuvalu	14.52	9.81	20.56	26.82	17.65	38.83	3.13	2.16	4.57
Uganda	50.85	35.14	68.19	70.46	48.10	93.72	34.65	23.36	51.84
Ukraine	104.91	74.82	140.15	173.05	112.76	248.67	55.03	36.98	80.32
United Arab Emirates	56.30	44.95	68.80	46.00	35.29	58.37	93.62	74.72	120.59
United Kingdom	86.55	82.41	89.96	117.19	112.02	121.83	58.59	54.95	61.56

Location	Both sexes			Male			Female		
	Value	95%UI (lower)	95%UI (upper)	Value	95%UI (lower)	95%UI (upper)	Value	95%UI (lower)	95%UI (upper)
United Republic of Tanzania	43.56	26.86	61.62	51.44	26.90	78.00	36.21	24.76	54.39
United States of America	75.11	70.92	78.25	109.40	103.70	113.72	44.81	41.50	46.99
United States Virgin Islands	64.33	49.53	83.43	103.42	75.44	137.96	28.87	19.26	44.69
Uruguay	170.16	153.31	186.61	261.61	230.15	297.36	95.87	82.35	109.90
Uzbekistan	42.24	32.47	53.48	55.09	41.68	70.70	31.43	23.84	39.95
Vanuatu	12.10	7.88	17.71	22.15	13.93	33.10	2.37	1.48	3.55
Venezuela (Bolivarian Republic of)	76.48	58.51	100.32	102.41	74.71	137.72	53.60	39.14	73.43
Viet Nam	14.17	11.08	17.94	22.71	17.35	29.21	7.30	5.01	9.87
Yemen	23.14	13.57	33.86	31.62	19.29	45.98	15.01	7.61	24.89
Zambia	66.67	31.02	105.60	94.64	36.41	151.33	39.76	22.80	72.95
Zimbabwe	40.86	30.01	53.19	47.31	36.10	61.24	36.15	24.39	50.06

UI uncertainty interval

Table S3 Age-standardized rate (ASR) of disability-adjusted Life years (DALYs) for prostate cancer (PCa) and testicular cancer (TCa) for all countries in 2021

Location	PCa (male)			TCa (male)		
	Value	95% UI (lower)	95% UI (upper)	Value	95% UI (lower)	95% UI (upper)
Global	217.83	192.65	235.53	13.83	13.03	14.73
Afghanistan	145.64	95.53	200.64	3.72	2.43	5.29
Albania	253.92	172.04	368.42	21.49	13.20	32.27
Algeria	46.25	29.09	63.26	2.97	2.04	4.32
American Samoa	858.56	679.20	1122.42	0.90	0.61	1.26
Andorra	368.01	244.70	545.13	12.45	7.99	19.11
Angola	486.19	296.55	656.44	13.60	9.53	18.91
Antigua and Barbuda	1259.86	1116.50	1423.65	10.21	8.84	11.70
Argentina	374.33	322.90	430.65	52.41	45.31	59.84
Armenia	301.79	254.92	346.75	16.17	11.67	21.61
Australia	351.29	297.40	411.89	14.83	12.61	17.67
Austria	304.91	256.34	358.18	13.40	11.24	15.99
Azerbaijan	182.46	130.05	245.22	9.76	5.29	16.52
Bahamas	1186.79	962.96	1454.10	2.79	2.24	3.54
Bahrain	331.41	238.60	441.83	3.44	2.43	4.65
Bangladesh	94.83	51.69	172.23	12.54	7.91	18.10
Barbados	867.79	668.36	1086.98	4.43	3.36	5.64
Belarus	433.51	329.57	568.40	16.09	12.58	19.95
Belgium	307.69	258.49	357.35	10.58	8.83	13.02
Belize	600.63	514.23	696.84	10.65	9.26	12.17
Benin	485.67	264.52	665.39	5.83	4.21	7.86
Bermuda	781.17	644.74	956.59	4.55	3.62	5.86
Bhutan	92.34	53.34	169.92	10.94	6.46	17.25
Bolivia (Plurinational State of)	504.23	325.38	753.15	36.00	22.07	54.67
Bosnia and Herzegovina	288.65	189.63	394.91	17.24	11.57	24.19
Botswana	763.86	516.89	1014.56	16.40	10.42	23.58
Brazil	374.97	347.39	399.19	24.53	22.48	26.40
Brunei Darussalam	228.10	163.81	292.41	13.93	9.31	19.57
Bulgaria	409.16	349.37	470.64	63.00	49.16	78.32
Burkina Faso	463.78	251.09	642.49	5.54	3.68	7.69
Burundi	405.12	201.27	622.03	12.07	7.35	19.30
Cabo Verde	898.17	538.70	1452.58	2.14	1.40	3.39
Cambodia	256.04	159.60	347.39	10.77	7.23	15.22
Cameroon	561.62	268.60	841.28	6.87	4.46	9.95
Canada	265.92	231.74	302.41	17.00	14.18	20.26
Central African Republic	451.66	252.09	647.76	10.87	6.97	16.80
Chad	446.17	233.77	658.63	6.26	3.98	9.36
Chile	392.22	330.19	456.08	68.62	56.86	80.91
China	76.97	56.59	103.60	5.56	4.32	7.01

Location	PCa (male)			TCa (male)		
	Value	95% UI (lower)	95% UI (upper)	Value	95% UI (lower)	95% UI (upper)
Colombia	337.92	274.15	410.90	27.95	22.82	33.20
Comoros	476.20	260.58	725.00	17.11	11.63	24.59
Congo	527.07	300.61	700.51	14.95	9.35	23.29
Cook Islands	1028.46	806.16	1313.43	3.26	2.14	4.63
Costa Rica	415.86	360.20	476.10	34.17	28.73	40.23
Coted'Ivoire	772.46	512.77	1049.54	6.76	4.40	9.37
Croatia	422.36	349.52	509.11	23.07	18.54	28.90
Cuba	705.22	598.15	834.42	14.79	12.20	17.87
Cyprus	382.22	286.88	491.29	12.54	9.27	16.64
Czechia	352.67	294.31	412.65	34.39	26.37	43.68
Democratic People's Republic of Korea	93.12	64.77	121.35	5.80	3.70	8.89
Democratic Republic of the Congo	444.24	276.84	642.27	11.22	6.88	16.63
Denmark	476.91	409.02	543.02	13.01	10.72	16.04
Djibouti	530.08	258.11	835.76	19.71	12.10	30.46
Dominica	1067.87	713.93	1495.06	5.65	3.99	7.95
Dominican Republic	531.21	338.22	875.08	1.77	1.13	2.59
Ecuador	373.55	292.48	470.76	27.27	21.68	33.99
Egypt	140.53	94.51	181.33	4.31	3.21	5.87
El Salvador	399.86	302.17	547.05	21.35	16.21	27.43
Equatorial Guinea	534.38	282.75	767.59	14.81	9.13	23.37
Eritrea	497.53	235.46	706.70	16.63	10.36	24.11
Estonia	652.76	511.91	805.03	13.96	11.53	16.58
Eswatini	812.86	479.28	1236.00	13.94	8.93	19.50
Ethiopia	154.62	83.80	229.74	14.91	7.42	21.31
Fiji	381.90	125.55	591.00	31.40	22.76	42.48
Finland	354.36	300.94	411.49	8.68	7.15	10.42
France	325.03	273.60	385.12	18.72	15.43	23.01
Gabon	587.39	312.59	854.33	14.97	9.86	23.32
Gambia	123.19	81.37	173.58	12.61	8.30	19.48
Georgia	523.21	450.67	608.88	63.03	50.33	78.22
Germany	349.38	298.50	400.79	18.84	16.05	22.11
Ghana	642.74	467.49	875.63	6.83	4.20	11.24
Greece	301.99	268.10	337.46	20.52	17.90	23.83
Greenland	215.48	158.52	284.55	19.21	12.69	27.60
Grenada	1542.79	1349.06	1750.86	22.23	19.10	25.94
Guam	239.73	191.00	329.06	4.35	3.59	5.26
Guatemala	383.25	325.30	454.33	31.67	27.00	36.44
Guinea	331.05	210.81	459.24	20.19	12.86	29.43
Guinea-Bissau	567.59	293.76	833.56	7.54	5.12	11.04
Guyana	938.06	704.53	1205.47	15.14	11.64	19.30
Haiti	902.52	565.27	1233.44	6.80	4.03	10.36

Location	PCa (male)			TCa (male)		
	Value	95% UI (lower)	95% UI (upper)	Value	95% UI (lower)	95% UI (upper)
Honduras	331.19	201.45	562.89	9.71	5.58	15.45
Hungary	342.87	282.83	403.94	43.40	34.49	53.94
Iceland	390.43	329.01	460.93	6.73	5.55	8.32
India	96.37	76.67	130.72	12.11	10.36	14.16
Indonesia	221.89	135.59	299.79	10.61	6.98	15.75
Iran (Islamic Republic of)	188.35	125.86	221.59	9.35	7.82	11.27
Iraq	126.86	86.58	181.51	6.43	4.26	9.39
Ireland	291.52	241.56	344.53	13.63	11.38	16.21
Israel	179.88	151.81	208.86	6.94	5.75	8.40
Italy	245.93	220.72	270.26	19.43	16.94	22.27
Jamaica	1003.43	753.18	1297.19	6.65	4.92	8.80
Japan	131.07	117.63	141.09	7.91	7.08	8.92
Jordan	134.39	85.79	185.14	10.38	7.11	15.07
Kazakhstan	194.30	165.86	224.83	23.43	16.18	31.98
Kenya	305.34	199.84	401.56	2.88	2.16	3.75
Kiribati	233.86	164.82	327.06	4.90	3.38	6.81
Kuwait	173.71	133.16	221.18	4.27	3.22	5.59
Kyrgyzstan	146.05	115.19	179.22	17.55	13.12	22.76
Lao People’s Democratic Republic	190.49	121.96	270.59	8.40	5.67	12.23
Latvia	623.04	481.35	767.49	31.59	25.62	38.64
Lebanon	313.05	207.39	416.02	10.46	7.30	14.53
Lesotho	841.33	546.11	1242.92	14.90	10.34	20.55
Liberia	440.17	222.12	656.42	5.80	3.43	9.08
Libya	243.61	139.44	360.43	3.01	2.06	4.47
Lithuania	596.24	478.97	728.61	18.69	15.45	21.99
Luxembourg	281.12	239.23	323.21	9.87	8.54	11.41
Madagascar	336.35	171.35	513.02	12.50	8.73	17.53
Malawi	399.40	284.98	538.30	29.94	20.29	43.53
Malaysia	183.43	121.43	234.58	14.24	11.30	17.68
Maldives	105.67	61.98	150.73	5.02	3.81	6.79
Mali	210.47	146.99	281.42	14.71	9.79	20.85
Malta	194.98	158.67	236.55	16.22	13.65	19.58
Marshall Islands	495.20	326.59	665.96	4.01	2.66	5.60
Mauritania	517.57	261.13	747.13	7.92	5.23	11.60
Mauritius	303.83	273.87	334.08	17.58	16.18	19.17
Mexico	310.54	261.50	367.08	68.52	61.24	76.56
Micronesia (Federated States of)	519.67	362.64	685.18	4.32	3.01	5.75
Monaco	418.68	309.42	589.88	72.83	45.29	110.28
Mongolia	85.97	61.49	114.38	17.61	12.96	23.38
Montenegro	523.71	387.72	702.00	42.67	29.72	57.54
Morocco	106.50	54.34	150.88	1.24	0.82	2.01

Location	PCa (male)			TCa (male)		
	Value	95% UI (lower)	95% UI (upper)	Value	95% UI (lower)	95% UI (upper)
Mozambique	198.72	142.46	283.42	17.47	10.68	26.76
Myanmar	188.79	126.32	261.01	7.16	4.83	10.02
Namibia	716.15	454.43	923.31	21.09	13.32	32.10
Nauru	541.85	338.74	835.74	4.01	2.94	5.57
Nepal	89.06	55.04	157.88	9.54	5.67	14.06
Netherlands	360.05	315.59	408.96	14.08	11.95	16.69
New Zealand	371.07	311.65	432.89	16.34	14.19	18.81
Nicaragua	277.72	201.00	378.07	19.06	12.08	28.93
Niger	403.32	202.51	642.19	4.82	2.48	7.94
Nigeria	802.83	357.38	1202.91	1.01	0.71	1.38
Niue	517.58	343.19	724.68	3.93	2.90	5.38
North Macedonia	299.20	200.81	400.52	43.11	31.99	55.29
Northern Mariana Islands	452.90	340.02	586.78	3.24	2.52	4.16
Norway	397.50	353.13	437.75	11.24	9.73	13.07
Oman	80.94	54.39	109.56	1.42	0.97	1.99
Pakistan	196.53	134.57	277.26	33.12	23.16	45.17
Palau	496.40	356.22	731.37	0.00	0.00	0.01
Palestine	318.24	248.00	453.72	7.16	5.43	9.66
Panama	381.93	294.30	475.20	20.21	16.55	23.90
Papua New Guinea	348.86	193.92	528.63	3.14	1.91	4.67
Paraguay	432.39	252.51	649.27	25.86	18.17	37.79
Peru	344.42	229.75	510.63	24.17	16.70	34.28
Philippines	293.35	213.25	391.44	11.09	8.91	13.66
Poland	444.24	395.26	492.58	41.88	37.64	45.66
Portugal	332.68	279.11	392.25	12.23	10.34	14.53
Puerto Rico	357.59	291.12	431.31	26.19	21.48	31.99
Qatar	354.99	229.67	526.75	2.22	1.48	3.19
Republic of Korea	124.04	75.33	159.89	2.69	1.92	3.67
Republic of Moldova	314.03	263.90	368.33	18.95	16.60	21.34
Romania	327.37	262.58	399.03	28.46	23.32	34.33
Russian Federation	374.01	330.42	416.77	20.65	18.87	22.51
Rwanda	500.79	268.34	739.36	15.27	10.23	22.31
Saint Kitts and Nevis	1517.58	1257.15	1810.32	17.21	12.89	23.81
Saint Lucia	1201.96	965.31	1442.34	23.19	19.19	27.58
Saint Vincent and the Grenadines	1325.19	1160.22	1491.68	15.07	13.23	17.16
Samoa	249.41	139.37	342.56	35.10	22.50	53.48
San Marino	213.71	139.89	332.24	7.79	4.50	12.21
Sao Tome and Principe	388.07	263.54	527.25	6.17	3.87	9.67
Saudi Arabia	87.16	57.10	163.55	4.63	3.00	7.05
Senegal	560.48	289.73	785.37	8.27	5.67	12.24
Serbia	339.00	230.36	450.09	29.75	18.55	45.43

Location	PCa (male)			TCa (male)		
	Value	95% UI (lower)	95% UI (upper)	Value	95% UI (lower)	95% UI (upper)
Seychelles	867.98	641.10	1088.19	11.61	8.53	14.95
Sierra Leone	440.97	238.45	652.06	5.75	3.55	8.18
Singapore	128.69	107.86	150.00	5.57	4.32	7.12
Slovakia	393.23	273.97	537.25	38.36	24.17	58.29
Slovenia	438.84	355.41	534.98	20.22	16.49	24.82
Solomon Islands	409.97	269.80	592.16	3.86	2.41	5.68
Somalia	388.51	182.48	631.57	11.03	4.54	19.89
South Africa	732.97	551.75	869.27	15.29	13.25	17.60
South Sudan	414.32	204.82	637.80	11.62	6.14	18.13
Spain	248.10	211.52	289.90	8.25	6.77	10.07
Sri Lanka	108.42	70.65	153.10	6.88	4.71	9.53
Sudan	141.54	94.23	207.09	4.61	3.00	6.87
Suriname	631.21	407.18	911.72	13.79	9.66	19.40
Sweden	386.51	319.37	455.36	7.39	6.08	8.96
Switzerland	326.48	271.95	382.26	8.43	6.96	10.27
Syrian Arab Republic	168.24	116.55	263.41	3.14	2.17	4.63
Taiwan (Province of China)	192.07	170.35	214.13	6.87	5.95	7.99
Tajikistan	78.15	48.91	122.07	0.73	0.39	1.20
Thailand	187.84	101.68	269.50	13.42	9.54	17.97
Timor-Leste	161.06	104.03	223.01	7.87	5.42	11.42
Togo	564.12	274.51	812.23	7.17	4.38	10.37
Tokelau	478.91	344.94	637.94	4.59	3.38	6.20
Tonga	633.14	484.29	829.01	3.84	2.72	5.54
Trinidad and Tobago	920.61	705.90	1162.05	11.94	9.07	15.53
Tunisia	131.53	75.00	190.31	4.24	2.86	6.34
Turkey	283.29	182.05	382.88	28.34	21.59	36.81
Turkmenistan	102.53	79.13	132.96	47.97	31.56	75.04
Tuvalu	463.65	337.86	603.10	3.70	2.62	5.16
Uganda	1003.78	744.03	1392.15	16.44	11.21	24.02
Ukraine	317.58	208.93	447.24	30.12	20.22	41.13
United Arab Emirates	222.81	160.79	329.20	9.19	6.75	12.33
United Kingdom	378.95	348.79	400.66	11.19	10.18	12.52
United Republic of Tanzania	432.13	218.61	637.01	16.12	11.45	22.18
United States of America	334.57	306.05	363.08	17.47	16.18	19.11
United States Virgin Islands	640.83	389.86	1074.22	3.32	2.08	4.76
Uruguay	590.15	504.13	687.75	52.81	43.34	63.38
Uzbekistan	87.69	72.22	104.36	19.91	14.52	27.44
Vanuatu	414.80	295.59	547.58	3.11	2.15	4.41
Venezuela (Bolivarian Republic of)	595.67	448.77	782.56	21.22	16.05	27.92
Viet Nam	64.20	32.70	86.23	9.00	6.22	12.89
Yemen	158.42	97.50	226.93	3.78	2.32	5.78

Location	PCa (male)			TCa (male)		
	Value	95% UI (lower)	95% UI (upper)	Value	95% UI (lower)	95% UI (upper)
Zambia	833.10	344.82	1321.84	51.98	31.93	78.89
Zimbabwe	1069.82	630.41	1390.23	13.52	9.03	19.13

UI uncertainty interval

Table S4 Summary indicators of sociodemographic index (SDI) related inequality in age-standardized rate (ASR) of disability-adjusted life years (DALYs) for four urinary tumors worldwide in 1990 and 2021

Diseases	Health inequality metrics	Year	Value	95% CI
BCa	Slope index of inequality	1990	76.07	59.08 − 93.06
		2021	110.57	94.41 − 126.73
	Concentration index	1990	0.36	0.31 − 0.42
		2021	0.38	0.33 − 0.43
KCa	Slope index of inequality	1990	57.31	45.45 − 69.17
		2021	105.97	90.41 − 121.53
	Concentration index	1990	0.41	0.36 − 0.47
		2021	0.39	0.34 − 0.44
PCa	Slope index of inequality	1990	329.90	251.20 − 408.60
		2021	544.03	455.31 − 632.75
	Concentration index	1990	0.44	0.36 − 0.52
		2021	0.31	0.24 − 0.37
TCa	Slope index of inequality	1990	14.45	9.96 − 18.94
		2021	7.32	3.25 − 11.4
	Concentration index	1990	0.21	0.16 − 0.26
		2021	0.06	-0.01 − 0.13

BCa bladder cancer, *KCa* kidney cancer, PCa prostate cancer, *TCa* testicular cancer, *CI* confidence interval

Table S5 The global trends in age-standardized rate (ASR) and case number of incidence for four urinary tumors between 1992 and 2021, and predicted changes to 2046

Year	BCa		KCa		PCa		TCa	
	**ASR (per 100,000 persons)**	**Case number**	**ASR (per 100,000 persons)**	**Case number**	**ASR (per 100,000 persons)**	**Case number**	**ASR (per 100,000 persons)**	**Case number**
1992	6.99	276,313	4.02	172,488	34.15	559,111	1.50	40,320
1993	7.05	284,963	4.12	180,119	34.92	587,034	1.51	41,388
1994	7.11	294,336	4.20	187,547	35.73	617,079	1.53	42,688
1995	7.16	302,827	4.26	193,597	36.32	644,576	1.59	45,060
1996	7.17	310,085	4.27	198,257	36.79	671,026	1.66	48,081
1997	7.15	316,217	4.29	202,974	36.66	687,026	1.67	49,222
1998	7.11	322,341	4.32	208,748	36.72	707,822	1.70	50,702
1999	7.11	330,340	4.33	214,083	36.61	725,968	1.72	52,046
2000	7.06	336,305	4.39	221,603	36.48	744,136	1.72	52,975
2001	7.02	342,607	4.44	229,405	36.63	768,904	1.70	53,209
2002	6.95	348,005	4.47	236,670	36.80	796,026	1.73	54,955
2003	6.91	354,417	4.53	245,456	36.90	821,761	1.74	56,048
2004	6.89	362,659	4.57	253,794	36.92	846,808	1.77	58,062
2005	6.92	373,904	4.67	265,445	37.17	878,488	1.81	60,157
2006	6.87	381,127	4.68	272,946	37.26	908,575	1.84	62,301
2007	6.82	389,497	4.69	281,023	37.23	936,865	1.87	64,504
2008	6.81	400,129	4.75	291,767	37.13	964,314	1.90	66,421
2009	6.77	408,797	4.77	301,050	36.81	987,219	1.94	69,050
2010	6.79	421,600	4.81	311,309	36.51	1,009,804	1.96	70,716
2011	6.74	430,695	4.80	319,110	36.06	1,029,177	1.97	71,860
2012	6.68	439,664	4.79	326,505	35.39	1,043,517	1.98	73,435
2013	6.62	448,770	4.78	334,660	34.63	1,053,767	2.02	75,543
2014	6.57	458,471	4.75	341,461	34.58	1,086,272	2.08	78,841

Year	BCa		KCa		PCa		TCa	
	ASR (per 100,000 persons)	Case number	ASR (per 100,000 persons)	Case number	ASR (per 100,000 persons)	Case number	ASR (per 100,000 persons)	Case number
2015	6.57	471,602	4.75	350,316	34.56	1,118,724	2.11	80,961
2016	6.57	485,562	4.72	357,530	34.71	1,159,506	2.13	82,776
2017	6.51	496,009	4.66	362,456	34.54	1,192,467	2.19	85,832
2018	6.45	506,171	4.63	369,684	34.52	1,230,990	2.25	89,255
2019	6.43	519,348	4.61	377,132	34.46	1,269,619	2.29	91,650
2020	6.36	528,203	4.53	379,894	34.14	1,293,915	2.23	90,300
2021	6.35	540,310	4.52	387,829	34.05	1,324,383	2.24	91,507
2022	6.23	539,202	4.48	390,409	34.02	1,320,733	2.35	97,637
2023	6.20	551,412	4.45	397,124	33.91	1,358,435	2.39	100,045
2024	6.17	565,309	4.42	404,655	33.81	1,400,584	2.42	102,516
2025	6.14	579,877	4.39	412,652	33.78	1,444,983	2.45	104,725
2026	6.12	594,563	4.37	420,564	33.74	1,489,392	2.48	106,948
2027	6.09	609,749	4.35	428,545	33.71	1,535,140	2.51	109,193
2028	6.06	625,636	4.32	436,749	33.67	1,583,703	2.54	111,471
2029	6.04	641,443	4.30	444,822	33.64	1,632,440	2.57	113,767
2030	6.02	657,580	4.28	453,300	33.66	1,682,765	2.59	115,708
2031	6.00	673,699	4.26	461,607	33.67	1,732,704	2.61	117,655
2032	5.98	690,353	4.25	469,939	33.69	1,783,513	2.63	119,638
2033	5.96	707,772	4.23	478,482	33.71	1,836,755	2.66	121,641
2034	5.94	724,991	4.21	486,860	33.72	1,890,045	2.68	123,629
2035	5.92	742,461	4.20	495,685	33.80	1,945,580	2.69	125,176
2036	5.91	759,582	4.19	504,250	33.87	2,000,416	2.70	126,700
2037	5.89	776,845	4.18	512,672	33.95	2,055,449	2.71	128,242
2038	5.88	794,703	4.17	521,177	34.02	2,112,297	2.73	129,775
2039	5.87	812,261	4.15	529,516	34.10	2,169,509	2.74	131,271
2040	5.86	830,035	4.15	538,162	34.21	2,228,775	2.75	132,648

Year	BCa		KCa		PCa		TCa	
	**ASR (per 100,000 persons)**	**Case number**	**ASR (per 100,000 persons)**	**Case number**	**ASR (per 100,000 persons)**	**Case number**	**ASR (per 100,000 persons)**	**Case number**
2041	5.85	847,247	4.14	546,522	34.32	2,287,393	2.76	133,979
2042	5.84	864,319	4.13	554,638	34.44	2,345,584	2.77	135,291
2043	5.83	881,802	4.12	562,678	34.55	2,404,133	2.78	136,566
2044	5.82	898,856	4.11	570,490	34.66	2,462,508	2.80	137,796
2045	5.81	915,305	4.11	578,075	34.77	2,520,835	2.81	138,971
2046	5.80	931,200	4.10	585,393	34.88	2,578,858	2.82	140,108

BCa bladder cancer, *KCa* kidney cancer, PCa prostate cancer, *TCa* testicular cancer
